# Resolving the mechanical paradox of myelination

**DOI:** 10.1038/s42003-026-10114-1

**Published:** 2026-04-28

**Authors:** Marco Fritzsche

**Affiliations:** 1 Kennedy Institute of Rheumatology, University of Oxford, Oxford, OX37FY United Kingdom; 2 Rosalind Franklin Institute, Harwell Campus, Didcot, OX11 0FA United Kingdom

## Abstract

Current preclinical models fail to capture the mechanics of oligodendrocyte myelination. Lasli et al.^1^ now demonstrate that the mechanical compliance of the axonal niche is a key determinant of oligodendrocyte maturation. By developing a platform that mimics the extreme mechanical softness of the central nervous system, they reveal myelination as a mechanically gated process as much as a biochemically regulated one.

The central nervous system is among the softest tissues in the body, yet our understanding of myelination has been built on a mechanical paradox. The complex choreography of oligodendrocyte (OL) differentiation and myelin wrapping has historically been studied on glass, plastic, or rigid polymer substrates that are mechanically several orders of magnitude stiffer than a living axon. On such giga- to mega- pascal-range substrates, OLs experience a persistent, non-physiological hard mechanical environment that can pre-drive maturation and mask subtle mechanosensation required for physiological myelination.

Lasli et al. now provide a micropillar-based platform that solves this problem^1^ by simultaneously controlling axon-mimetic geometry and soft-tissue mechanics. Their findings expose that OL wrapping around an axon is not just a biochemically regulated signalling process but a mechanically gated decision (Fig. 1).

**Fig. 1:**
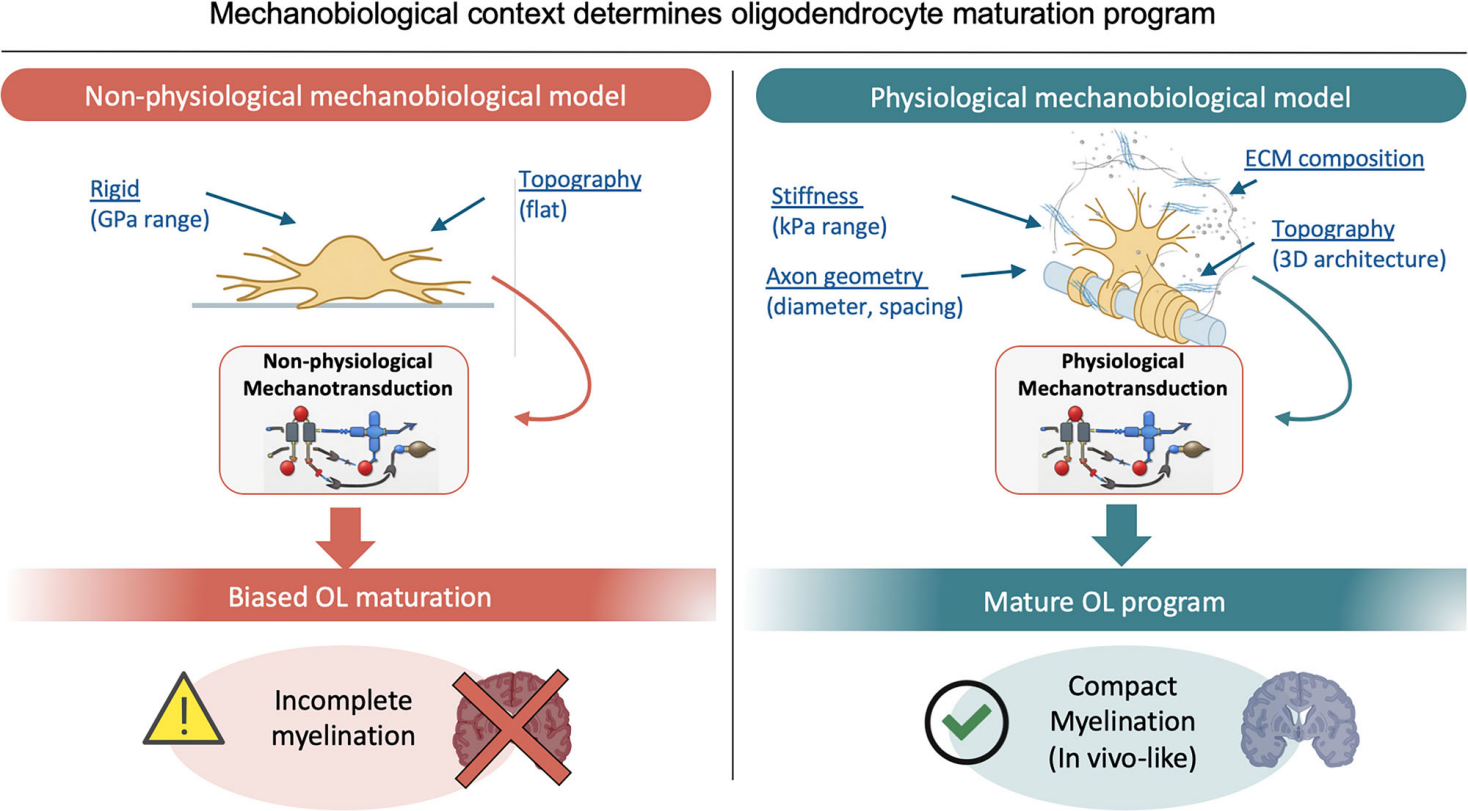
Mechanobiological fidelity governs the OL maturation program. Schematic illustrating how the physical microenvironment shapes glial fate. Left: Traditional rigid substrates (GPa range) create a “mechanical mismatch” that triggers aberrant, constitutive mechanotransduction, biasing OLs towards a non-physiological maturation state. Right: A biomimetic 3D hydrogel platform integrates brain-like stiffness (kPa range) and axon-mimetic geometry^1^. This physiological niche supports physiologically relevant mechanosensitive signalling, enabling the study of the fundamental physical thresholds required for multilayered myelin wrapping and homeostatic glial function.

Understanding OL wrapping requires identifying how OLs sense axonal mechanical compliance, likely through force-sensitive receptors and cytoskeletal reorganisation that translate mechanical cues into signalling for glial wrapping^2^. However, as we attempt to quantify the mechanosensation of OL wrapping, we must navigate the “mechanobiologist’s dilemma”^3^. When living OLs adapt to the very soft mechanical properties of the pillars, changes in their own cellular mechanics can alter the sensitivity of the very tools used to measure them. Decoupling the biological response from the inherent technical measurement sensitivities will be essential to ensuring we observe true mechanosensation rather than an artifact of measurement.

The most significant contribution of this study is the shift from a “cell-centric” to a “niche-centric” view of myelination. By establishing that the mechanical properties of the axonal environment act as a gatekeeper for glial fate, we can begin to reframe remyelination failure, particularly in chronic multiple sclerosis lesions, not merely as a loss of cellular potency, but as a loss of niche receptivity. If the local tissue environment undergoes pathological mechanical softening or structural degradation, it may create a physical barrier that renders even the most potent pro-myelinating drugs ineffective. Consequently, the “active” niche described by Lasli et al. suggests that future regenerative strategies may need to be bimodal: targeting the intrinsic biology of the OL while simultaneously “priming” the mechanical landscape of the axon to lower the threshold for repair.

This work arrives as remyelination failure is increasingly understood as a problem of tissue context as much as cell-intrinsic biology^4^. In conditions like multiple sclerosis and ageing, changes in tissue compliance may create a mechanical barrier to remyelination that is as consequential as canonical molecular inhibitors. In academia, Lasli’s technology enables systematic dissection of how mechanical signals are integrated with functional axonal activity, providing a blueprint for the “active” niche. In pharma, the hydrogel-based micropillar arrays provide a scalable, high-content imaging platform for drug screening. By supporting the formation of compact, multilayered myelin from human sources, this approach reduces our reliance on animal models and mitigates the high rate of “false-positive” hits.

Although further limitations remain, such as the need to integrate with axonal electrical activity, the authors’ innovative mechanosensitive platform thus provides a springboard for studying myelin mechanobiology and a roadmap for remyelinating therapies for neurodegenerative diseases like multiple sclerosis.
